# Quo vadis, LDL cholesterol?

**DOI:** 10.1515/almed-2023-0051

**Published:** 2023-05-31

**Authors:** Andrés Cobos, Pedro Valdivielso

**Affiliations:** UGC de Laboratorio, Hospital Universitario Virgen de la Victoria, Málaga, Spain; Servicio de Medicina Interna, Hospital Universitario Virgen de la Victoria, Departamento de Medicina y Dermatología, Universidad de Málaga e Instituto de Investigación Biomédica de Málaga (IBIMA-Plataforma BIONAND), Málaga, Spain

**Keywords:** basic lipid profile, LDL cholesterol, Spanish Consensus

The prevention of cardiovascular disease requires controlling risk factors, including LDL cholesterol, an etiologic risk factor. In the recent years, evidence has been provided that levels of LDL cholesterol (LDLc) should be measured and used instead of total cholesterol as a reference in therapeutic decision making. Fortunately, this evidence has been widely accepted and considered not only by clinical guidelines, but also by health professionals. Not long ago, access to HDL cholesterol (HDLc) values was limited, since this parameter was only measured when abnormalities were observed in other parameters, or in the hospital setting.

However, these limitations have disappeared, and scientific advances have made it possible to determine a wide range of lipid parameters. The information reported by clinical laboratories should not only meet physician’s request, but they should also be based on scientific evidence and be clinically relevant. On such purpose, the Multidisciplinary Task Group on Lipids and Vascular Risk, composed of representatives from 13 Spanish scientific societies, presents on this Issue a “Consensus Document on Lipid Profile Testing and Reporting in Spanish Clinical Laboratories”, [1].

This Consensus Document provides a description of key aspects of lipid testing, both in the pre-analytical (fasting or not, patient position during blood collection, clinical status of the patient) and post-analytical phase. The Consensus identifies the basic parameters that should be included in what they call “basic profile”, which includes serum concentrations of total cholesterol, triglycerides, HDL cholesterol, LDL cholesterol (estimated), and non-HDL cholesterol. This basic information helps clinicians assess vascular risk and verify the degree of control of dyslipidemia in a patient. Also, the basic profile helps determine whether the required therapeutic target has been achieved. Very elevated or very low values of cholesterol, triglycerides, or HDL cholesterol should raise suspicion of monogenic dyslipidemia [2]. Another relevant aspect mentioned on the Consensus is that laboratories should inform clinicians that hyperlipidemia, apart from being a risk factor, can be a disease itself. Judiciously, the Consensus Document recommends laboratories to include Dutch Lipid Clinical Network Score (DLCNS) for familial hypercholesterolemia, or Moulin score on suspicion of familial chylomicronemia syndrome based on the presence of severe hypertriglyceridemia.

The Consensus also discusses some basic aspects of LDLc determination, which gold standard method, density-gradient ultracentrifugation, is known to be only available in clinical research laboratories. On the understanding that the estimation of LDLc is influenced by values of triglycerides, and based on the evidence available, the Consensus proposes three formulas: the traditional Friedewald formula, Martin–Hopkins formula, and Sampson formula. These three methods for the estimation of LDLc have been compared not only in terms of triglycerides, but also in terms of reliability in very low LDLc concentrations, which are achieved through the triple therapy [3]. Nevertheless, there is evidence that the three formulas can be used indistinctly [4]. This situation leads to inaccuracy in the measurement of a key parameter in cardiovascular prevention [5], which may be misleading to clinicians. The Consensus highlights two alternatives for the estimation of LDLc: non-HDL cholesterol and apolipoprotein B, being the former the recommended one, as it involves no cost. But that’s not all. The most recent ESC 2021 guidelines [6] recommend using non-HDLc instead of total cholesterol or total cholesterol/HDLc rate to estimate the risk for a fatal event at 10 years. In addition, these new guidelines consider non-HDLc a therapeutic target (30 mg/dL higher than then recommended LDLc). The question is: if non-HDLc testing is inexpensive, useful for risk assessment (as it comprises the cholesterol carried by all atherogenic particles), and is a therapeutic target, why is LDLc not removed from the focus of cardiovascular prevention and replaced with non-HDLc?

Beyond some basic considerations, the Consensus suggests measuring cholesterol in remnant particles, although this parameter is influenced by LDL cholesterol. Measuring remnant particles or cholesterol in remnant particles provides information on the vascular risk of a patient [7], especially in cases of mixed dyslipidemia or hypertriglyceridemia. However, since this parameter has a poor clinical value [8], it is not included in the basic profile.

Of special note, Lp (a) is included in the basic profile (tested at least once in life), but not in routine testing until a specific medication is designed for this lipoprotein. Strikingly, it is recommended that a flag should be included when Lp (a) values exceed 120 mg/dL. This cutoff value may seem very elevated, as values>50 mg/dL increase vascular risk substantially in patients with familial hypercholesterolemia [9]. In addition, in the trial with Pelacarsen (Horizon), two cutoff points were established for allocation to one of the two arms: >70 mg/dL and >90 mg/dL. However, these levels are consistent with the recommendations of the Spanish Society of Atherosclerosis for referral of patients with Lp (a) >117 mg/dL [10] to a Lipid Unit, which is based on a population study conducted in Copenhagen [11].

The Consensus also incorporates the inclusion of desirable LDL cholesterol according to the vascular risk of the patient, instead of using “normality” values. It is also recommended to include flags that warn about critical values, which will lead the clinician to order further studies or refer the patient to a specialty unit. Obviously, the inclusion of flags would result in an increase in referrals to reference units. This complication could be overcome through e-consultations. Telemedicine is defined as medical care provided by a health professional to a patient using electronic communications technology, which spares face-to-face visits. In contrast, teleconsultation is defined as a synchronous or asynchronous consultation by which health professionals share information, thereby overcoming functional or geographical distance [12]. In this sense, the Consensus Document lists 11 components that should be included in e-consultations related to vascular risk. Teleconsultations among health professionals will progressively evolve, at least in the hospital setting, to benefit both, health professionals, and patients, thereby reducing the traditional gap between levels of healthcare. In Spain, e-consultations are available for patients with heart failure and atrial fibrillation [13], which enables the optimization of face-to-face medical care planning.

Finally, the Consensus provides extensive supplementary material that compiles technical information about analytical variation, clinical and pharmacological causes of dyslipidemia, and questionnaires for familial hypercholesterolemia and familial chylomicronemia syndrome.

It is a valuable document that will help clinical laboratories and clinicians share and receive very useful information for the prevention of vascular disease and diagnosis of dyslipidemias.
